# D-mannose ameliorates autoimmune phenotypes in mouse models of lupus

**DOI:** 10.1186/s12865-020-00392-7

**Published:** 2021-01-05

**Authors:** Haiting Wang, Xiangyu Teng, Georges Abboud, Wei Li, Shuang Ye, Laurence Morel

**Affiliations:** 1 Department of Rheumatology, RenJi Hospital South Campus, School of Medicine, Shanghai Jiaotong University, Shanghai, China; 2 Department of Pathology, Immunology and Laboratory Medicine, University of Florida, JHMHC 275, Box 100275, Gainesville, FL 32610-0275 USA

**Keywords:** Systemic lupus erythematosus, D-mannose, T cells, Dendritic cells

## Abstract

**Background:**

Systemic lupus erythematosus is an autoimmune disease characterized by an overproduction of autoantibodies resulting from dysregulation in multiple immune cell types. D-mannose is a C^− 2^ epimer of glucose that exhibits immunoregulatory effects in models of autoimmune diseases, such as type 1 diabetes, induced rheumatoid arthritis, and airway inflammation. This study was conducted to evaluate the efficacy of D-mannose treatment in mouse models of lupus.

**Results:**

Firstly, the effect of D-Mannose was evaluated by flow cytometry on the in vitro activation of non-autoimmune C57BL/6 (B6) bone marrow-derived dendritic cells (BMDCs) and their ability to induce antigen-specific CD4^+^ T cell proliferation and activation. D-mannose inhibited the maturation of BMDCs and their induction of antigen-specific T cell proliferation and activation. In vivo, D-mannose increased the frequency of Foxp3^+^ regulatory T cells in unmanipulated B6 mice. To assess the effect of D-mannose in mouse models of lupus, we used the graft-versus-host disease (cGVHD) induced model and the B6.lpr spontaneous model. In the cGVHD model, D-mannose treatment decreased autoantibody production, with a concomitant reduction of the frequency of effector memory and follicular helper T cells as well as germinal center B cells and plasma cells. These results were partially validated in the B6.lpr model of spontaneous lupus.

**Conclusion:**

Overall, our results suggest that D-mannose ameliorates autoimmune activation in models of lupus, at least partially due to its expansion of Treg cells, the induction of immature conventional dendritic cells and the downregulation of effector T cells activation. D-Mannose showed however a weaker immunomodulatory effect in lupus than in other autoimmune diseases.

**Supplementary Information:**

The online version contains supplementary material available at 10.1186/s12865-020-00392-7.

## Background

Systemic lupus erythematosus (SLE) is an autoimmune disease with a diverse clinical presentation. Tissue damage is mediated by pathogenic autoantibodies, however, abnormalities in cell development and function are not limited to B cells. Indeed, most types of immune cells are involved in SLE pathogenesis [1]. Among them, autoreactive CD4^+^ T cells, which have altered signaling and functions, play an essential role in several disease processes [2]. Lupus CD4^+^ T cells also present a skewed metabolic program that is directly associated with these immune dysregulations [3]. Simultaneous administration of the mitochondrial metabolism inhibitor metformin and the glucose metabolism inhibitor 2-deoxy-D-glucose (2DG) ameliorated disease severity in several strains of lupus-prone mice, which strongly suggests that manipulating metabolism in lupus may offer novel therapeutic options [4].

D-mannose, a C-2 epimer of glucose, has a physiological blood concentration of less than 2% of that of glucose [5]. However, when imported into cells, D-mannose interferes with the metabolism of glucose [6]. In CD4^+^ T cells, D-mannose decreases glycolysis and promotes fatty acid oxidation (FAO), which is associated with regulatory T (Treg) cell suppressive phenotypes [7]. Indeed, D-mannose promoted the expansion in the frequency and the function of Treg cells in vitro and in vivo [8]. Accordingly, administration of D-mannose in drinking-water suppressed the immunopathology of autoimmune diabetes and airway inflammation in mouse models [8]. However, it is unknown whether the administration of D-mannose could suppress immunopathology in models of lupus.

Lupus development is associated with changes in the dendritic cell (DC) compartment, including an altered frequency and localization of DC subsets, overactivation of conventional (cDCs) and plasmacytoid (pDC) DCs, as well as functional defects [9]. The mannose receptor (MR, also known as CD206) is an endocytic receptor involved in antigen presentation. The expression of CD206 on DCs impaired CD8^+^ T-cell cytotoxicity by direct interaction with CD45, and up-regulation of CTLA on the T cells [10]. In murine collagen-induced arthritis, blocking CD206 attenuated disease by modulating the differentiation, maturation, and functions of DCs, which shifted CD4^+^ T cell polarization from a Th1 / Th17 to Th2 cell [11]. It remains unclear whether D-mannose treatment in drinking water could regulate DCs function in a similar fashion by promoting CD206 functions. Here, we show that D-mannose enhanced the expression of CD206 and Fcγ receptor in bone marrow-derived dendritic cells (BMDCs), which are markers characteristic of immature DCs, and decreased their ability to activate CD4^+^ T cells.

We assessed the effects of D-mannose treatment in the chronic graft-versus-host disease (cGVHD) model, a well characterized induced model of lupus [12] as well as in the spontaneous B6.lpr model. We have shown a beneficial effect of the inhibition of glycolysis in both of these models {Yin 2015; Yin 2016; Li 2018}, which supports the hypothesis that D-mannose could also impact their autoimmune pathogenesis outcomes. Each model presents specific advantages to assess the effect of treatment of the autoimmune system. Induced cGVHD provides a rapid, predictable autoimmune activation with low variability. In addition, it allows the study of the effect of treatments in both donor T cells and host B cells and DCs, which all play an essential role in cGVHD response. B6.lpr mice spontaneously develop an SLE-like phenotype, in which autoimmunity arises from a completely different pathway compared with cGVHD. This model displays increased numbers of CD3^+^CD4^−^CD8^−^ T cells and an enhanced Th17: Treg ratio. T We showed that D-mannose alleviated CD4^+^ T cell activation, expanded the frequency of Treg cells, restrained DC activation and ameliorated lupus phenotypes. These results suggest an immunoregulatory effect of D-mannose on DC and T cell phenotypes in lupus models.

## Results

### D-mannose inhibited BMDC activation and BMDC-induced antigen-specific T-cell responses in vitro

**Fig. 1:**
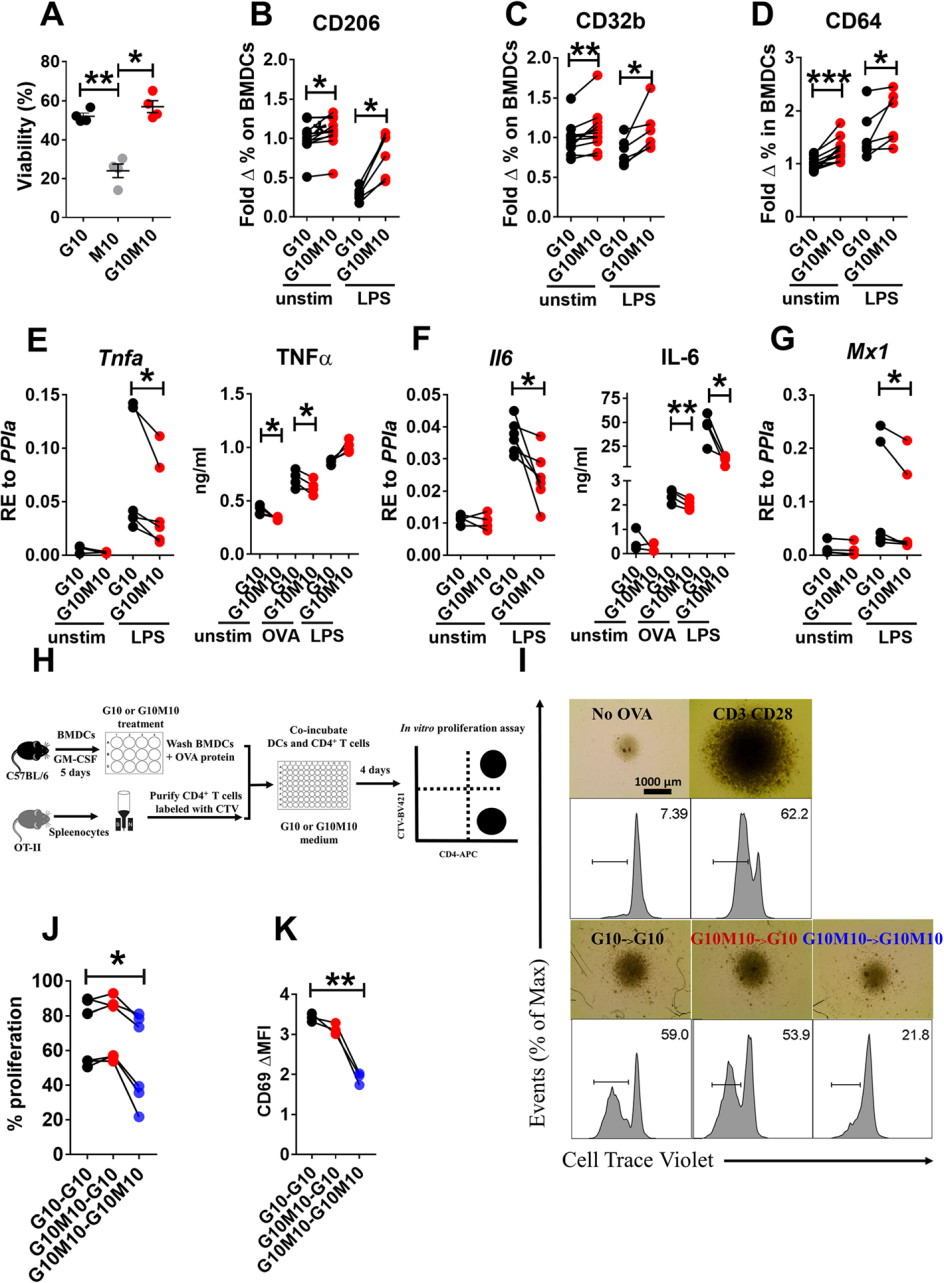
D-mannose inhibits BMDCs activation *in vitro* and BMDC-induced antigen-specific T-cell responses in vitro*.* BMDCs from B6 mice were cultured with G10, M10, or G10M10 for 24 h with or without LPS, or with OVA. Cell viability (**a**) and surface marker expression (**b**-**d**) were analyzed by flow cytometry. TNF*-α* (**e**) and IL-6 (**f**) were measured at the message level in unstimulated and stimulated cells and the protein level in the supernatants. (**g**) *Mx1* gene expression was measured in unstimulated and stimulated cells. **h**. B6 BMDCs were treated with G10 or G10M10 medium for 24 h, then loaded with OVA protein and incubated with OT-II CD4^+^ T cells in G10 or G10M10 co-culture medium for 4 d. At d5, proliferation (**i** and **j**) and CD69 expression (**d**) were measured in CD4^+^ T cells by flow cytometry comparing 3 conditions: G10 for both BMDCs and co-culture (G10 – G10), G10M10 BMDCs and G10 co-culture (G10M10 – G10), or G10M10 for both BMDCs and co-culture (G10M10 – G10M10). Mean ± S.E.M of *n* = 4–7 per group from 2 to 3 separate experiments compared with t tests. *: *p* < 0.05, **: *p* < 0.01, ***: *p* < 0.001

An in vitro study was carried out to evaluate the effect of D-mannose relative to glucose on the function of BMDCs. The viability of BMDCs greatly decreased after a 24 h culture in a glucose-free culture medium with 10 mM D-mannose alone (Fig. 1a). The addition of D-mannose to glucose did not affect DC viability (Fig. 1a). Since a glucose-free condition is not physiologically relevant, we compared 10 mM glucose (G10) to the combination of 10 mM glucose and D-mannose (G10M10) in the culture media. D-Mannose enhanced the expression of the mannose receptor CD206 (Fig. 1b) whether or not the BMDCs were activated with LPS. The expression of two other surface receptors that regulate DC functions, CD32b (FcγRIIb), an inhibitory FCγ receptor, and CD64 (FcγRI), an endocytic receptor involved in antigen uptake [13], were also increased by the addition of D-mannose, even in the presence of LPS stimulation (Fig. 1c-d). Notably, this effect of D-mannose on BMDCs was observed without altering the expression of activation markers (CD40, CD80 and CD86, data not shown). Finally, the production of pro-inflammatory cytokines, IL-6, TNF-α and type 1 IFN (detected through the expression of the interferon stimulated gene *Mx1*), was decreased at the mRNA level after D-mannose treatment in LPS-activated BMDCs, but no difference was observed in unstimulated BMDCs (Fig. 1e-g). At the protein level, unstimulated BMDCs and BMDCs pulsed with OVA produced lower amounts of TNF-α in the presence of D-mannose (Fig. 1e). Furthermore, OVA-pulsed and LPS-stimulated BMDCs produced lower amounts of IL-6 (Fig. 1f).

In light of the critical role of DCs in priming T cells, we next investigated the effect of D-mannose on the antigen-presenting function of OVA-loaded BMDCs co-cultured with OVA-specific OT-II CD4^+^ T cells (Fig. 1h). The proliferation of T cells, assessed by CTV dilution, was suppressed when both BMDCs and BMDC:T cell co-cultures were exposed to D-mannose, but not when the BMDCs alone were loaded with OVA in the presence of D-mannose (Fig. 1i - j). The same result was obtained for the expression of CD69, an early T cell activation marker (Fig. 1k). Although D-mannose may also have an effect on T cells themselves, which cannot be experimentally assessed in an antigen-specific context, our findings suggest that D-mannose dampens antigen-specific activation of T cells by DCs. This may due to D-mannose inducing an immature, anti-inflammatory phenotype in DCs that inhibits antigen-specific T cell proliferation and activation during antigen presentation.

### D-mannose expanded the steady-state Treg population in non-autoimmune B6 mice

**Fig. 2:**
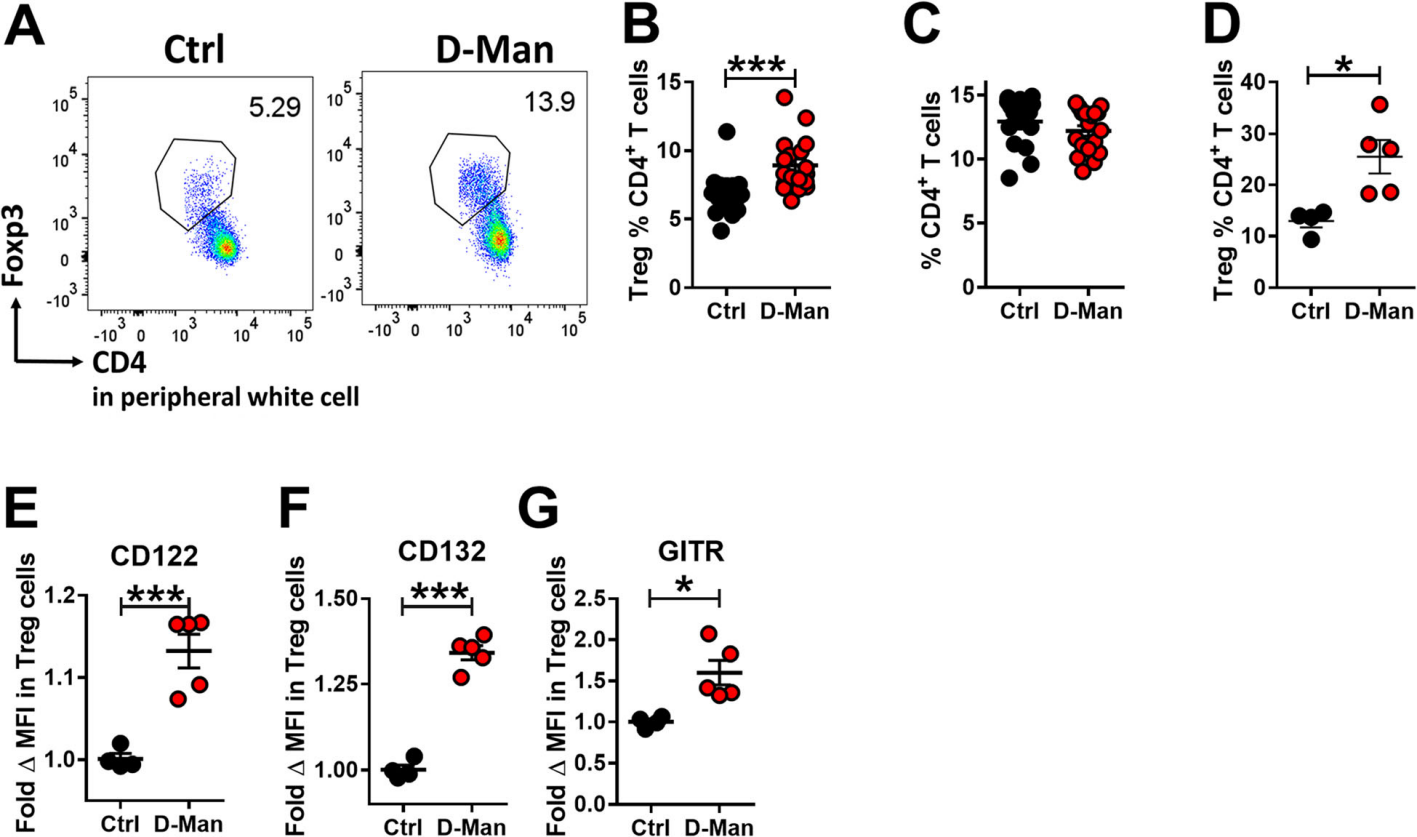
D-mannose expanded the steady-state Treg population. B6 mice were treated with or without D-mannose in drinking water. Phenotypes were analyzed at week 3 in the peripheral blood and week 9 in the spleen. **a**. Representative FACS plots of Treg cells in CD3^+^-gated PBMCs. Frequency of Treg cells (**b**) and CD4^+^ T cells (**c**) in CD3^+^ PBMCs. *n* = 16 per group from 2 separate experiments compared with t tests. **d**. Frequency of Treg cells in CD4^+^ splenocytes**.** CD122 (**e**), CD132 (**f**) and GITR (**g**) MFI on Treg cells expressed as fold change relative to the Ctrl group. Mean ± S.E.M of *n* = 4–5 per group compared with t tests. *: *p* < 0.05, ***: *p* < 0.001

In vivo, we first tested the effect of D-mannose in unmanipulated B6 mice treated for up to 9 weeks. At week 3, the frequency of circulating CD4^+^ Foxp3^+^ Treg cells was higher in D-mannose-treated mice (Fig. 2a and b), while the frequency of total CD4^+^ T cells remained unchanged (Fig. 2c). After 9 weeks of treatment, the frequency of Treg cells was increased in the spleen of D-mannose treated-mice (Fig. 2d). Moreover, the expression of Treg-associated receptors, including CD122, CD132 (the IL-2Rβ and γ chains, respectively), and GITR, was increased in D-mannose treated mice (Fig. 1 e - g). No difference was, however, observed for the expression of CD25, the IL-2Rα high affinity chain (data not shown). These results showed that D-Mannose expanded the Treg population and the expression of some of its markers under homeostatic conditions.

### D-mannose treatment decreased autoimmune activation in the chronic graft-versus-host disease (cGHVD) model

**Fig. 3:**
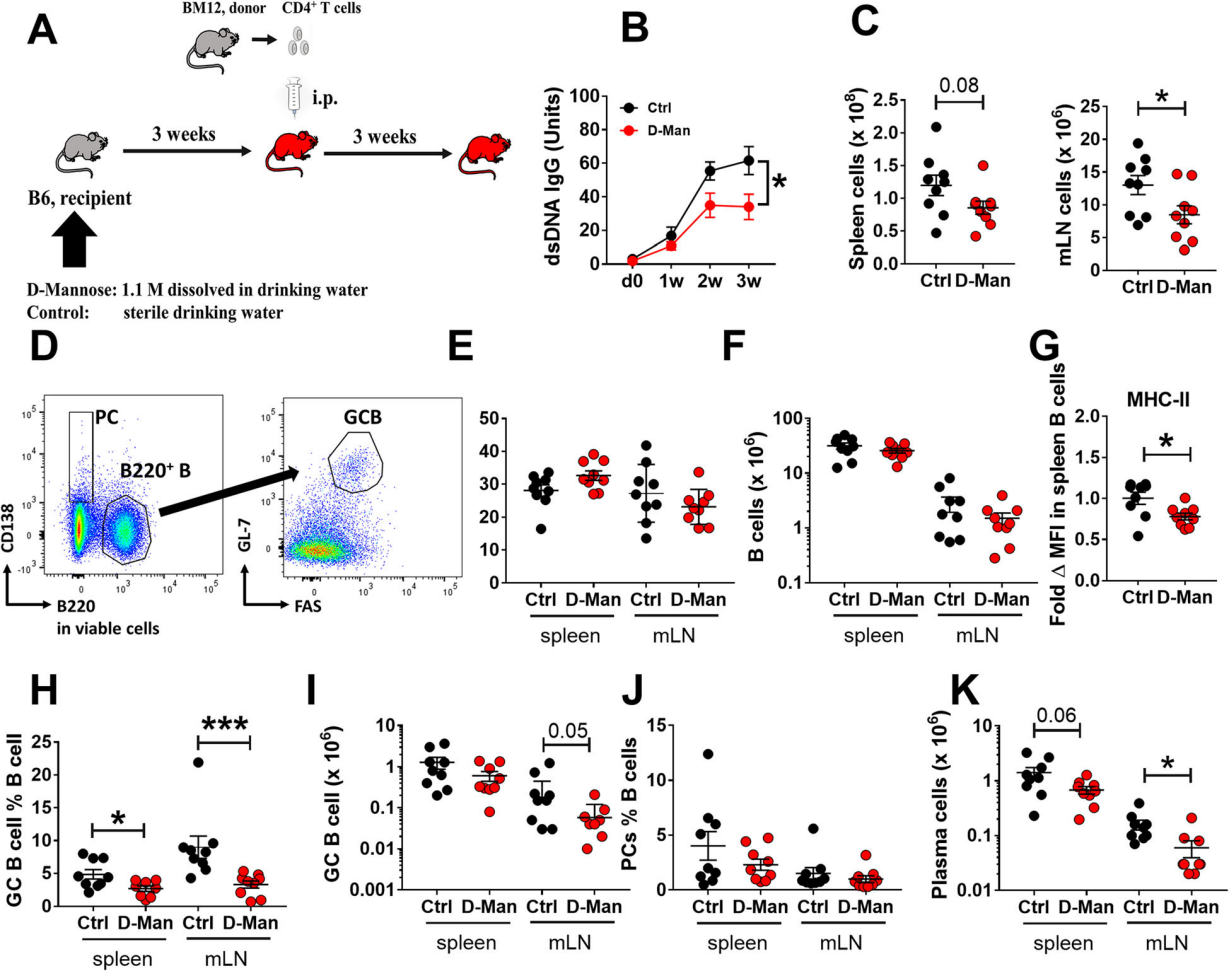
D-mannose reduced autoantibody production, lymphoid expansion as well as B cell differentiation in the cGVHD model. **a**. Experimental design. **b**. Serum anti-dsDNA IgG. **c**. Representative FACS plots of B cells showing the GC B cell and plasma cell gates. Spleen and mLN cell numbers. Frequency (**e**) and number (**f**) of B cells in spleen and mLN. **g**. MHC-II MFI on splenic B cells expressed as fold change relative to the Ctrl group. Frequency and number of GC B cells (**h** and **i**) and plasma cells (**j** and **k)** in spleen and mLN. Mean ± S.E.M of *n* = 9–12 per group from 2 to 3 cohorts compared with t tests. *: *p* < 0.05

We next investigated the effect of D-mannose in the cGVHD model, a well-established model of induced systemic autoimmunity [12]. After 3 weeks of pre-treatment with or without D-mannose, B6 recipients received CD4^+^ T cells from the semi-allogenic bm12 donor, and continued their treatment with or without D-mannose for another 3 weeks (Fig. 3a). Mice were sacrificed 3 weeks after induction, which corresponds to the peak of anti-dsDNA IgG production in this model [14]. D-mannose reduced anti-dsDNA IgG production starting 2 weeks after induction (Fig. 3b). This suppressive effect was associated with a lower number of cells in the mesenteric lymph node (mLN), which is the lymphoid organ first impacted by oral delivery, and a similar trend was observed in the spleen (Fig. 3c). The frequency and number of splenic B cells remained unchanged (Fig. 3d - f), but their activation measured as the expression of MHC-II was decreased in D-mannose treated mice (Fig. 3g). D-mannose also decreased the frequency of germinal center (GC) B cells in both spleen and mLN, but cell numbers decreased only in mLN (Fig. 3h - i). There was also a reduction in the number of plasma cells of mLN and a similar trend in the spleen (Fig. 3j - k).

**Fig. 4:**
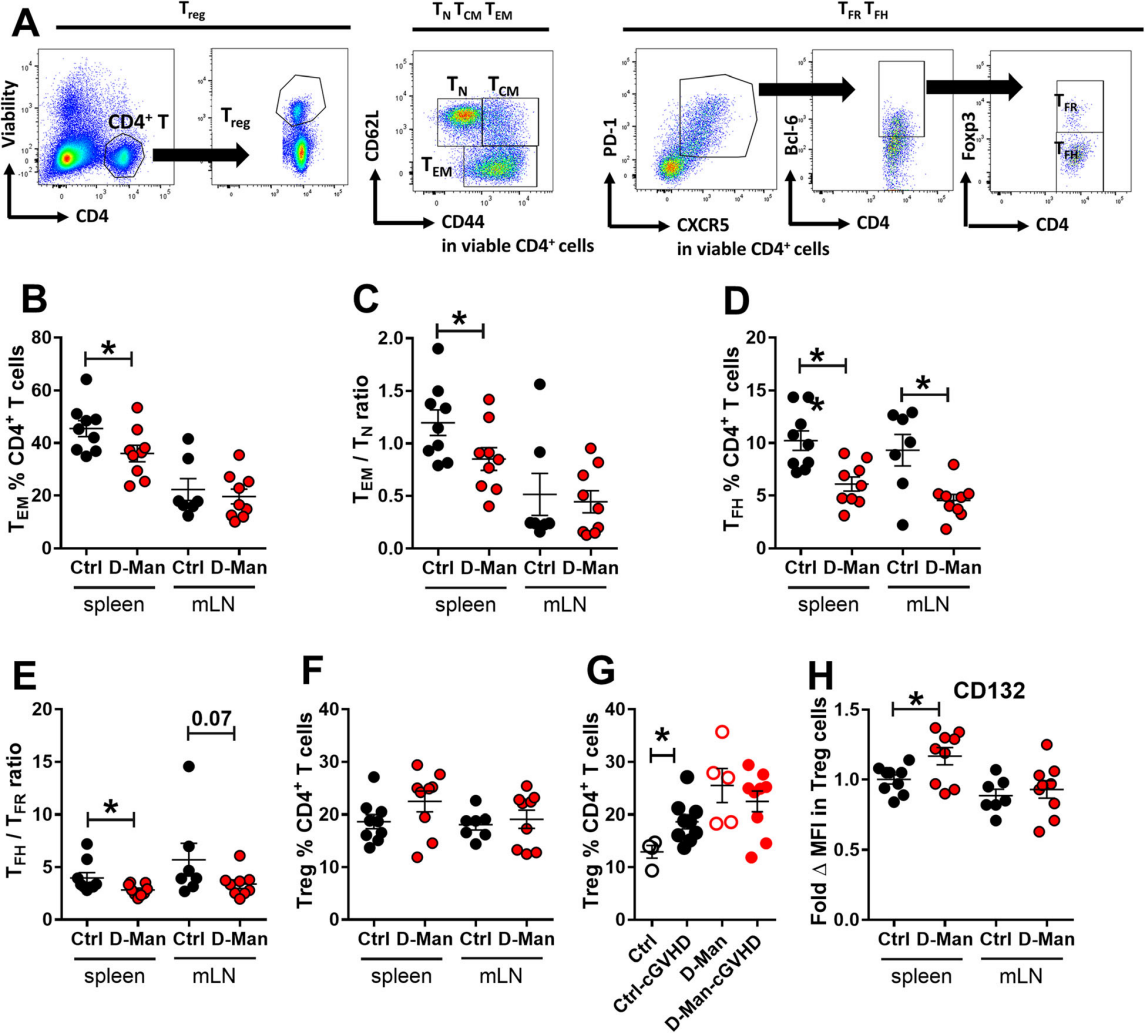
D-mannose reduced the expansion of effector CD4^+^ T cells in the cGVHD model. **a**. Representative FACS plots of CD4^+^CD44^−^CD62L^+^ T_N,_ CD4^+^CD44^+^CD62L^−^ T_EM,_ CD4^+^PD-1^+^CXCR5^+^Bcl-6^+^Foxp3^+^ T_FR_ and CD4^+^PD-1^+^CXCR5^+^Bcl-6^+^Foxp3^−^ T_FH_ cells, and CD4^+^Foxp3^+^ Treg cells 3 weeks after cGVHD induction. **b**. Frequency of T_EM_ cells. **c**. T_EM_/T_N_ ratio. **d**. Frequency of T_FH_ cells. **e**. T_FH_/T_FR_ ratio. **f**. Frequency of Treg cells. **g.** Comparison of the effect of D-mannose on splenic Treg cell frequency in unmanipulated mice (Ctrl) or cGVHD-induced mice. **h**. CD132 MFI on Treg cells expressed as fold change relative to the Ctrl group. Mean ± S.E.M of *n* = 9 per group from 2 to 3 cohorts compared with t tests. *: *p* < 0.05, **: *p* < 0.01

CD4^+^ T cell activation has been a major focus of investigation in the SLE-cGVHD model [15]. D-mannose treatment did not change the frequency or number of total CD4^+^ T cells (data not shown), but alleviated CD4^+^ T cell activation measured as the frequency of CD44^+^CD62L^−^ effector memory (T_EM_) cells (Fig. 4a - b), as well as the T_EM_ over naive CD44^−^ CD62L^+^ (T_N_) ratio in spleen (Fig. 4c). In addition, the frequency of follicular helper (T_FH_) T cells and the ratio of T_FH_/follicular regulatory (T_FR_) in spleen were also reduced after D-mannose treatment (Fig. 4d - e). Contrary to the results observed in non-inflammatory conditions (Fig. 2), the frequency of Treg cells was not altered by D-mannose in cGVHD-induced mice (Fig. 4f). However, cGVHD expanded the frequency of Treg cells in control mice, most likely as a response to inflammation, but not in D-mannose treated mice (Fig. 4g). In addition, a higher expression of the IL-2 receptor gamma chain (CD132) was observed in the splenic Treg cells from the D-mannose treated group in both cGVHD-induced mice (Fig. 4h) and in unmanipulated mice (Fig. 2f).

**Fig. 5:**
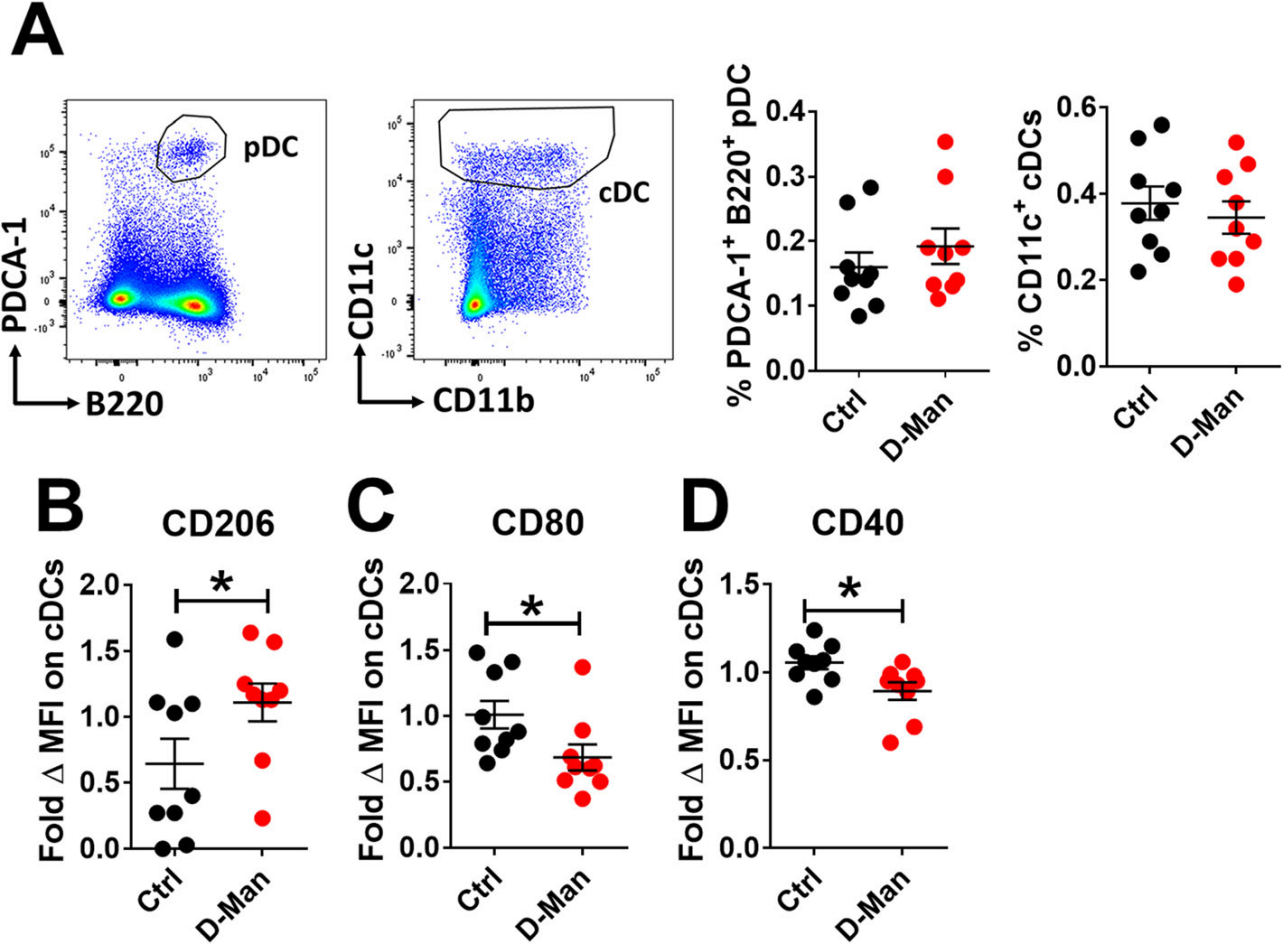
D-mannose reduced cDC activation. **a**. Representative FACS plots showing B220^+^PDCA-1^+^ pDC and CD11c^+^CD3^−^ cDC gating, as well as their number and frequency in the spleen of cGVHD-induced mice. CD206 (**b**)**,** CD80 (**c**), and CD40 (**d**) MFI on these cDCs expressed as fold change relative to the Ctrl group. Mean ± S.E.M of *n* = 9 per group from 2 to 3 cohorts compared with t tests. *: *p* < 0.05, **: *p* < 0.01

Finally, we determined the effect of D-mannose on DCs by assessing their frequency and the expression of activation markers. The frequency of plasmacytoid DCs (pDCs) and conventional dendritic cells (cDCs) was comparable in D-mannose treated and non-treated mice (Fig. 5a). however, the splenic cDCs from D-mannose treated mice displayed an increased expression of the mannose receptor (Fig. 5b) and a decreased expression of the activation markers CD80 and CD40 (Fig. 5c - d). Overall, these results suggest that D-mannose limits the development of autoimmune activation in cGVHD model.

### D-mannose decreased the number of T_EM_ and T_FH_ cells and expanded the frequency of Treg cells in cGVHD recipients

**Fig. 6:**
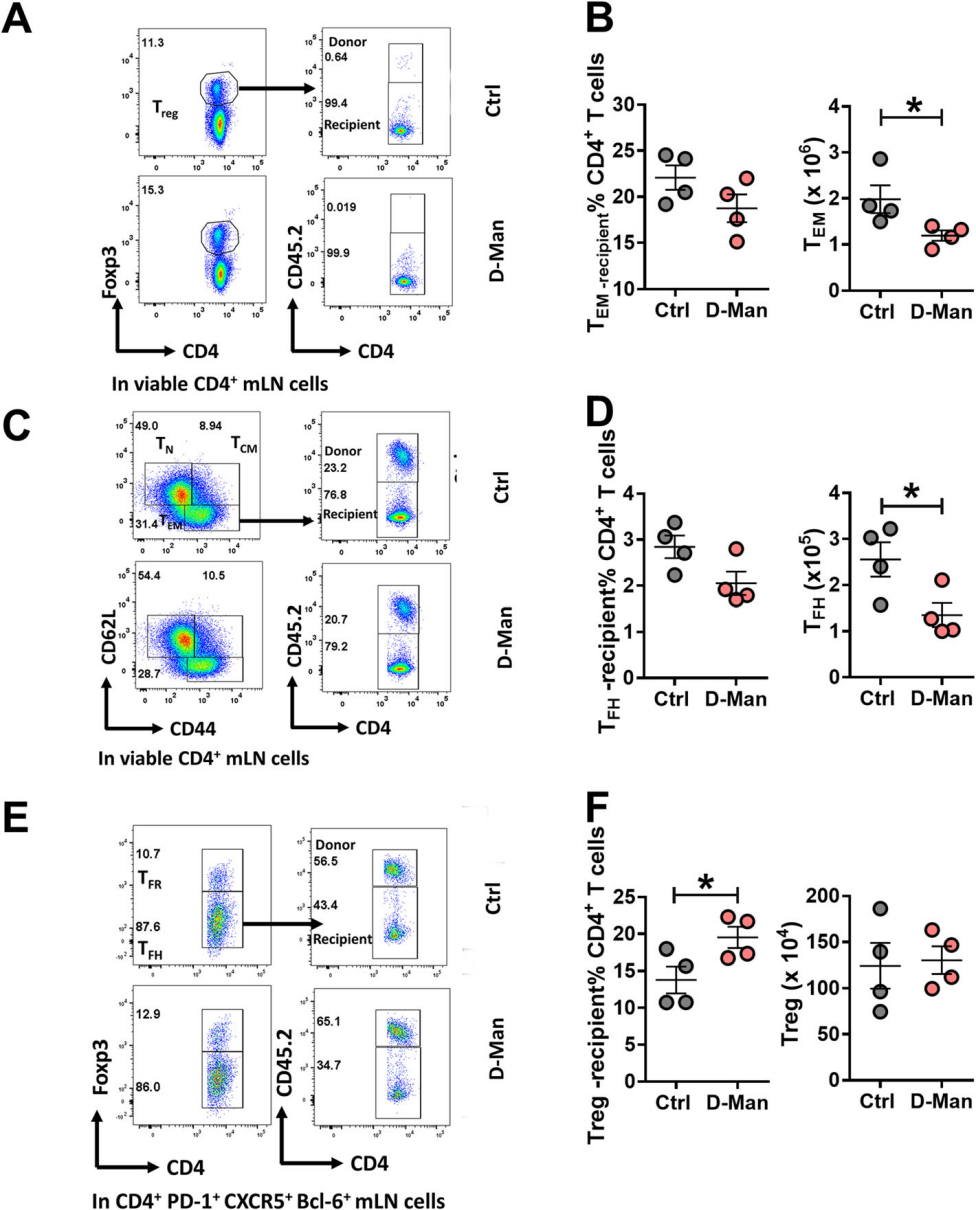
D-mannose decreased the number of T_EM_ and T_FH_ cells and increased the frequency of Treg cells in recipient mice in the cGVHD model. Frequency and numbers of recipient T_EM_ (**a**-**b**), T_FH_ (**c**-**d**) and Treg (**e**-**f**) cells in the mLN. Representative FACS plots of donor (CD45.2^+^) and recipient (CD45.2^−^) cells are shown on the left. Mean ± S.E.M of n = 4 per group compared with t tests. *: *p* < 0.05, **: *p* < 0.01

To differentiate the effect of D-mannose on donor or recipient CD4^+^ T cells in cGVHD, we used B6.SJL as recipients, in which we can distinguish recipient CD4^+^ T cells (CD45.1^+^ B6.SJL) from the donor CD4^+^ T cells (CD45.2^+^ − bm12). D-mannose affected CD4^+^ T cells in recipient mice, first by decreasing the frequency and number of recipient-derived T_EM_ (Fig. 6a – b) and number of recipient T_FH_ cells (Fig. 6c – d). Moreover, D-mannose increased the frequency of recipient Treg cells (Fig. 6e – f). It should be noted that less than 1% of the donor CD4^+^ T cells recovered after cGVHD expressed Foxp3 while over 80% of these donor cells expressed a T_EM_ phenotype (data not shown). These results confirm the immunoregulatory effect of D-mannose in the cGVHD model and showed that it does not change the inflammatory phenotypes of the donor cells, but the response in recipient cells.

### D-mannose treatment decreased CD4^+^ T cell activation in B6.Lpr mice

**Fig. 7:**
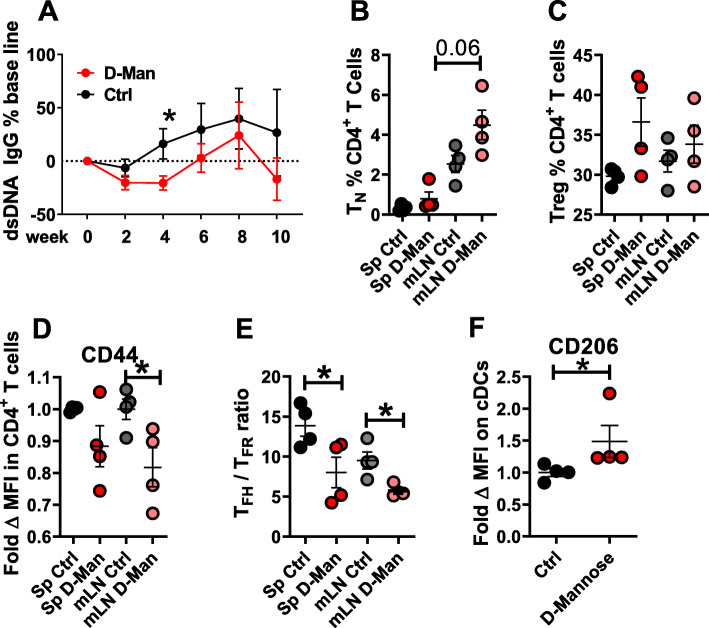
D-mannose ameliorates lupus phenotypes in B6/lpr mice. Four month old B6.lpr mice were treated with D-mannose in drinking water for 10 weeks. **a**. Serum anti-dsDNA IgG. Frequency of T_N_ (**b**) and Treg cells (**c**). **d**. CD44 MFI on CD4^+^ T cells. **e**. T_FH_ /T_FR_ ratio in spleen and mLN. **f**. CD206 MFI on spleen cDCs. Mean ± S.E.M of *n* = 4 per group compared with t tests. *: *p* < 0.05

To assess the efficacy of D-mannose in a spontaneous model of lupus, we selected B6.lpr mice, a simplified model driven by FAS deficiency. Treatment was started at 4 months of age, when B6.lpr mice start to produce autoantibodies, and lasted for 10 weeks. There was an initial reduction of anti-dsDNA IgG production, which reached significance at week 4 after treatment, but it was not sustained thereafter (Fig. 7a). There was a trend for an increase of the frequency of T_N_ cells (Fig. 7b) and Treg cells (Fig. 7c) by the D-mannose treatment. CD4^+^ T cell activation, measured as CD44 expression (Fig. 7d) and the T_FH_/T_FR_ ratio, (Fig. 7e) was reduced by the D-mannose treatment. Finally, splenic cDCs from D-mannose treated mice expressed higher levels of CD206 (Fig. 7f), which was in line with the result obtained in the cGVHD model. The D-mannose treatment did not affect the number and distribution of cDC1 and cDC2 subsets (data not shown). These results showed that the immunoregulatory effect of D-mannose is not restricted to the cGVHD induced model of lupus, but also applies to the B6.lpr model, in which autoimmunity arises from a completely different pathway.

## Discussion

The purpose of this study was to evaluate whether the protective effect of D-mannose that has been reported for type 1 diabetes, airway inflammation [8], and in an induced model of arthritis [11] could be extended to models of systemic autoimmunity. D-mannose treatment has not been tested in lupus, either in vitro or in vivo. Furthermore, the potential involvement of CD206, the mannose receptor, in the regulation of immune cells in SLE is largely unknown. CD206 is a marker of M2 macrophages, which are generally associated with anti-inflammatory phenotypes. Activated CD206^+^ M2b macrophages have been shown, however, to be pathogenic in a spontaneous model of lupus nephritis [16] and they represent the largest myeloid infiltrates in human lupus nephritis [17, 18]. On the other hand, CD206 expression on macrophages was induced by a therapeutic treatment with mesenchymal stem cells in an IL-6 dependent manner in the B6.lpr model [19]. Whether the expression of CD206 on these pro- or anti-inflammatory macrophages is necessary for their function is unknown.

We started our study with two cell types in which D-mannose has shown to modulate functions, DCs and Treg cells. Blocking the MR modulated the differentiation, maturation, and functions of DCs, reducing the severity of induced autoimmune arthritis [11]. Moreover, deleting or blocking CD206 on DCs decreased the proliferation and cytotoxic activity of urine CD8^+^ T cells in vitro [10]. The expression of surface molecules on DCs is dynamic and changes according to tissue localization and maturation state. Most immature DCs are characterized by expression of molecules associated with antigen uptake such as CD32b and CD64, but also CD206, which is an endocytic receptor. With activation, mature DCs lose their capacity for antigen engulfment, and acquire molecules necessary for stimulation of T lymphocytes, such as CD40, CD80, and CD86 [20]. In this study, we showed that D-mannose enhanced the expression of CD206 on cDCs both in vitro (BMDC) and in vivo (cGVHD and lupus-prone B6/lpr model). In addition, CD32b and CD64 expression remained high in D-mannose treated BMDCs in spite of LPS stimulation. These results indicate that D-mannose maintains DCs in an immature state despite activation, which is consistent with the finding that cross-linking CD206 induced anti-inflammatory DCs [21]. Here we showed that this result can be achieve directly with D-mannose.

Moreover, D-mannose treatment inhibited DC activation as shown in vitro by the reduced expression of pro-inflammatory cytokines and in the cGVHD model by a lower expression of the activation markers CD40 and CD80, which mediate interactions with T cells. Accordingly, D-mannose inhibited antigen specific proliferation and activation in CD4^+^ T cells in a co-culture assay. This inhibitory effect could not be detected when antigen uptake occurred in the presence of D-mannose, but without D-mannose in the co-culture medium. This result could be interpreted in two manners: first, the presence of D-mannose may be critical to maintain DCs in an immature state during antigen presentation; or second, D-mannose may directly induce the differentiation of Treg cells, which may synergize with immature DCs to suppress T cell proliferation and activation.

D-mannose promoted the expansion of Treg cells from naive CD4^+^ T cells in spontaneous type 1 diabetes as well as in a TCR transgenic model of induced airway inflammation [8]. We confirmed this effect of D-mannose in unmanipulated B6 mice, and further showed an induction of markers commonly associated with regulatory functions in Treg cells, namely, GITR, CD132, and CD122. In the cGVHD model, D-mannose upregulated the expression of CD132 on Treg cells, but it did not change Treg frequency or number three-week after induction. It is likely, however, based on our results obtained at steady state, that the three-week pre-treatment of the cGVHD recipients initially expanded their Treg population, which contributed to the lower cGVHD induction in these mice. There was a trend for an increased frequency of Treg cells in D-mannose treated B6.lpr mice, which may suggest that the induction of regulatory markers of Tregs may be more critical than the expansion of Treg number. Lupus cytokine milieu promotes the expansion of non-functional Treg cells [22] that may not respond to D-mannose as efficiently as control Treg cells. In support of this hypothesis, we showed that the frequency of Treg cells is higher in cGVHD-induced mice than in controls.

Nonetheless, we have shown that D-mannose treatment reduced autoantibody production in the cGVHD model of systemic autoimmunity as well as, at least at early age, in B6.lpr mice. This was associated with a reduction of CD4^+^ T cell differentiation into T_EM_ cells and T_FH_ cells, as well as the T_EM_/T_N_ cells and T_FH_/T_FR_ ratios. These effector T cell phenotypes are strongly associated with lupus pathogenesis in both mouse models and human patients [23]. Donor CD4^+^ T cells are both necessary and sufficient for cGVHD induction, but no effect of D-mannose could be detected in donor derived CD4^+^ T cell subpopulations, including Treg, T_EM_, or T_FH_ cells. However, there were significantly less recipient-derived T_EM_ and T_FH_ cells in the D-mannose treated group. The results suggest the expansion of the donor activated T cells was globally limited in the presence of D-mannose, and that they induced a reduced response in recipient T cells. Interestingly, more than 90% of Treg cells were of recipient origin, which was much higher compared with the percentage of T_EM_ and T_FH_ cells of recipient origin. As mentioned above, it is likely that these Treg cells as well as recipient DCs were modulated by D-mannose and played a significant role in the modulated phenotypes. Notably, the effect of D-mannose treatment was overall stronger in the mLN than in the spleen. The intraperitoneal induction of cGVHD combined to the oral delivery of D-mannose may be responsible for this tissue distribution. The underlying mechanism of the preferential effects on gut-associated recipient T cells warrants further investigation.

## Conclusions

We have shown an immunoregulatory effect of D-mannose in an induced lupus model, which was associated with the expansion of Treg cells, the induction of immature conventional dendritic cells and the downregulation of effector T cells activation. D-mannose had a more potent effect in type 1 diabetes and OVA-induced airway inflammation [8] than what we observed here in induced and spontaneous models of lupus. This may be related to differences in the pathogenic process between organ-specific T-cell mediated diseases in which Treg cells play a critical role and a systemic antibody-mediated disease in which Treg cells play a relatively reduced role. Therefore, the strong expansion of the Treg population mediated by D-mannose may have only a limited beneficial effect in the lupus models. Our findings warrant further exploration of the basic immunological mechanisms and potential clinical applications of D-mannose in various types of autoimmune / inflammatory diseases.

## Methods

### Mice

C57BL/6 J (B6) mice, B6(C)-*H2*-*Ab1*^*bm12*^/KhEgJ (bm12), B6.CgTg (TcraTcrb) 425Cbn/J (OT-II), B6.SJL-*Ptprc*^*a*^ *Pepc*^*b*^/BoyJ (B6.SJL) and B6.MRL-*Fas*^*lpr*^/J (B6.lpr) mice were obtained from the Jackson Laboratory. All mice were bred and maintained at the University of Florida in specific pathogen-free conditions. Both males and females were used between 2 and 6 months of age. Gender and age were matched for each experiment. D-mannose (Sigma) was dissolved at 1.1 M in drinking water and administered ad lib.

### cGVHD induction and analysis

cGHVD was induced according to an established protocol [12]. Briefly, 7–9 × 10^6^ CD4^+^ T cells isolated by negative selection (Miltenyi) from spleen and mLN of bm12 mice were transferred intra-peritoneally into B6 or B6.SJL mice. bm12 CD4^+^ T cells are sufficient to initiate cGVHD in B6 mice [24], which allowed us to focus on the effect of D-mannose on donor CD4^+^T cells instead of mixed cell populations from the whole spleen. Serum was collected weekly up to 3 weeks after induction, at which point mice were sacrificed to assess immunophenotypes.

### Flow cytometry

Single-cell suspensions were prepared from spleens and mLN using standard procedures. After RBC lysis, cells were stained in staining buffer (2.5% FBS, 0.05% sodium azide in PBS). Fluorochrome-conjugated Abs purchased from BD Biosciences, eBioscience, and BioLegend with the following specificities were used: CD3e (145-2C11), CD4 (RM4–5 and GK1.5), CD8α (53–6.7), CD11b (M1/70), CD11c (HL3), CD19 (6D5), CD25 (PCX61 and PC61.5), CD32b (AT130–2), CD40 (3/23), CD44 (IM7), CD45.2 (104), CD62L (MEL-14), CD64 (X54–5/7.1), CD69 (H1.2F3), CD80 (16-10A1), CD86 (GL1), CD95 (15A7), CD122 (TM-b1), CD132 (TUGm2), CD138 (281–2), CD206 (C068C2), B220 (RA3-6B2), Bcl-6 (K112–91), CXCR5 (2G8), Foxp3 (FJK-16S), GITR (DTA-1), GL-7 (GL-7), IgD (217–170), Ki-67 (16A8), PD-1 (RMP1–30), PDCA-1 (927), and MHC-II (M5/114.15.2). CD4^+^ follicular helper T (T_FH_) cells were stained as previously described [4] in a three-step process using purified CXCR5 (2G8) followed by biotinylated anti-rat IgG (Jackson Immunoresearch) and PerCP5.5-labeled streptavidin. Dead cells were excluded with fixable viability dye (eFluor780; eBioscience). Data were collected on LSRFortessa (BD Biosciences) and analyzed with FlowJo (Tree Star) software. IFNγ, IL-10 and IL-17A production were analyzed in cells treated with the leukocyte activation cocktail (BD Biosciences) for 5 h and the Fixation/Permeabilization kit (eBioscience). To avoid batch effect, mean fluorescence intensity (MFI) was reported as fold ΔMFI, defined as the MFI value in each sample divided by the mean MFI value in all controls. Similarly, the percent expression of makers in BMDC cultures was reported as fold Δ% defined as the percentage in each sample divided by the mean percentage in control BDMCs treated with 10 mM glucose (G10).

### Generation of BMDCs and BMDC/T cell co-cultures

Bone marrow single cell suspensions from B6 mice were cultured in RPMI-1640 supplemented with 10% FBS and 20 ng/mL of GM-CSF (R&D systems) for 5 d. Half of the media was replenished on d3. On d5, the medium was changed to complete glucose-free RPMI-1640 with 10% FBS, supplemented with 10 mM D-mannose (M10), 10 mM glucose (G10), or their combination (G10M10) for 24 h. The concentration of 10 mM glucose corresponds to that of standard culture medium, and it is close to the physiological concentration in normal human blood (5 mM). A concentration of 10 mM D-mannose is close to the in vivo pharmalogical concentration after acute D-mannose injection and it is also consistent with other reports {Zygmunt}. In some experiments, LPS (0.1 μg/mL) or ovalbumin (OVA, 30 μg/ml, Sigma) were added to the medium for 24 h on d5. For OVA-specific proliferation assays, M10, G10 or G10M10 treated BMDCs (5 × 10^4^) were pulsed with 30 μg/ml OVA for 2 h in complete RPMI-1640. Then, these BMDCs were incubated with Cell Trace Violet (CTV, Invitrogen Life Technologies) labeled OT-II CD4^+^ T cells (5 × 10^5^) in either G10 or G10M10 medium. After 96 h of culture, T cell proliferation was measured as the CTV dilution by flow cytometry.

### Antibody measurements

Serum autoantibody levels were determined as previously described by ELISA [25]. Briefly, anti-dsDNA IgG was measured in 1:100 diluted sera in duplicates and in plates coated with 50 μg/mL dsDNA (Sigma). The secondary antibody was goat anti-mouse IgG conjugated to alkaline phosphatase (Southern Biotech).

### Cytokine measurements

RNA was isolated from M10, G10 or G10M10-treated BMDCs with the RNeasy Mini Kit (Qiagen). qRT-PCR was performed with ImProm II Reverse Transcriptase (Promega) and SYBR Green Dye (Bio-RAD) on the BioRad CFX connect system as previously described [26] with primer sequences shown in Sup. Table 1. The PCR thermo-cycling protocol was 30 s at 95 °C, 30 s at 60 °C, and 30 s at 72 °C repeated for 42 cycles. Expression was calculated using the 2^ΔCq^ method with difference in Cq values normalized to the housekeeping gene *Ppia* against the genes of interest. TNF-α and IL-6 were measured in the culture supernatants with ELISA kits from BD OptEIA.

### Statistical analysis

Differences between groups were evaluated by two-tailed statistics: unpaired or paired t tests, or Mann-Whitney U tests depending on whether the data was normally distributed. The results are expressed as means ± S.E.M. The statistical analyses were performed with the Graphpad Prism 7.0 software. The level of statistical significance was set at * *p* < 0.05, ** *p* < 0.01, *** *p* < 0.001.

## Supplementary Information

**Additional file 1.**

## Data Availability

The datasets used and analyzed during the current study are available from the corresponding author on reasonable request.

## Abbreviations

D-man: D-mannose
cGVHD: Chronic graft-versus-host-disease
Treg: Regulatory T cells
GC: Germinal center
T_FH_: Follicular helper T cells
T_FR_: T follicular regulatory cells
T_N_: Naïve T cells
Teff: Effector T cells
SLE: Systemic lupus erythematosus
