# Supplementary figures and images for: D-mannose ameliorates autoimmune phenotypes in mouse models of lupus
Source: BMC Immunol. 2021 Jan 5;22:1. doi: 10.1186/s12865-020-00392-7 (PMC7786459; doi:10.1186/s12865-020-00392-7)

**Supplemental Table 1. Primers used for qRT-PCR.**

**
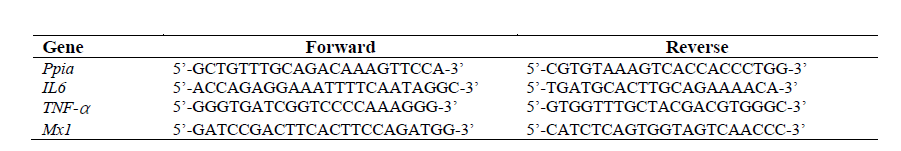
**

Supplement: Supplementary file 1 — Additional file 1. [file 12865_2020_392_MOESM1_ESM.docx]
